# Evaluating artificial intelligence-generated multiple-choice questions in clinical pathology

**DOI:** 10.1016/j.acpath.2026.100275

**Published:** 2026-06-17

**Authors:** A. Aziz Ould Ismail, Weijie Ma, Nancy M. Dunbar, K. Aaron Geno, Jeremiah X. Karrs, Isabella W. Martin

**Affiliations:** Department of Pathology and Laboratory Medicine, Dartmouth Hitchcock Medical Center, Lebanon, NH, USA

**Keywords:** Artificial intelligence, Assessments, Clinical pathology, Pathology education

## Abstract

Large language models are increasingly applied in medical education, but their role in clinical pathology remains uncertain. We conducted a prospective evaluation during the 2024–2025 academic year using GPT-4 with a standardized prompting protocol to generate multiple-choice questions for pathology resident education in clinical chemistry, hematopathology, clinical microbiology, molecular pathology, bioinformatics, and transfusion medicine. Faculty experts scored items for clarity and accuracy on a 1–5 scale and assigned a disposition of kept as is, kept but edited, or discarded. Of 260 items reviewed, 108 (41.5%) were retained without revision, 86 (33.1%) with edits, and 66 (25.4%) were discarded. Subspecialty performance varied, with molecular pathology showing the highest retention (60% unedited), while clinical microbiology had a relatively high discard rate (41.7%). Items retained without revision demonstrated significantly higher clarity and accuracy scores than discarded items (*P* < 0.05), and quality metrics also varied significantly across subspecialties (*P* < 0.001). Higher clarity (odds ratio*:* 1.68, 95% confidence interval*:* 1.12–2.54, *P* = 0.013) significantly predicted favorable disposition. Faculty comments revealed that AI-generated questions most often failed due to inappropriate difficulty calibration, lack of board relevance, and a disconnect from real-world laboratory practices. Reviewers favored practical, application-focused content over factual recall. Nearly three-quarters of artificial intelligence-generated questions were usable after expert review, highlighting the potential of large language models to accelerate assessment development. However, uneven subspecialty performance and risks of regulatory misalignment underscore the necessity of sustained expert oversight.

## Introduction

Medical education is undergoing rapid transformation with the integration of artificial intelligence (AI). Large language models (LLMs), such as OpenAI's GPT series, have demonstrated strong text-generation capabilities and are increasingly applied in educational settings^1^^,^^2^ including grading and adaptive learning.^3^^,^^4^ In medical training, where assessment item development is time-intensive and expertise-driven, AI tools offer potential efficiency gains while maintaining educational rigor.^5^ However, their role in specialized domains remains underexplored.

Clinical pathology—including clinical chemistry, hematopathology, clinical microbiology, molecular pathology, bioinformatics, and transfusion medicine—is among the most technically complex medical specialties. Practitioners not only interpret laboratory results but also apply diagnostic algorithms and understand testing methodologies ranging from microscopy to molecular assays. Unlike some specialties centered on patient-facing decisions, pathology education integrates laboratory science, diagnostic reasoning, and regulatory considerations.

Assessment frameworks in clinical pathology must therefore address multiple competencies. Trainees are expected to demonstrate factual understanding of disease, interpret results accurately, apply quality-control principles, understand regulatory requirements, and integrate laboratory findings with clinical scenarios. Assessment items often include detailed clinical scenarios, requiring significant expert input to ensure accuracy, realism, and curricular alignment.^6^ Clinical pathology assessments also require frequent updates. The field evolves rapidly, with new biomarkers, methods, and diagnostic standards regularly emerging. These advances force frequent content revisions, creating bottlenecks in curriculum development.

AI has been explored as a way to meet these challenges. Studies in medicine, pharmacology, and surgery show that AI can generate clinically relevant questions, though expert oversight is essential for nuance and accuracy.^7^ Pathology presents even greater challenges,^8^ as content requires precise terminology, laboratory methods, and subspecialty-specific integration of clinical and diagnostic knowledge.^9^ For example, clinical microbiology items emphasize organism identification and resistance patterns, whereas molecular pathology requires knowledge of genetic testing algorithms and variant interpretation.

The potential of AI in this space depends on the quality of its outputs. Poorly designed questions can propagate misconceptions, while high-quality items may ease faculty workload, expand assessment banks, and improve adaptability of curricula.^6^ Evaluation of AI outputs requires systematic expert review including checks for accuracy, clarity, technical correctness, and alignment with learning objectives.^10^

This study addresses this gap by systematically evaluating the ability of an LLM to generate multiple-choice questions (MCQs) in clinical pathology. We analyze item quality across subspecialties, identify common error types, and estimate levels of expert revision required. The findings provide evidence on how AI can be responsibly integrated into pathology education and may inform applications in other specialized domains of medical training.

## Materials and methods

### Study design and setting

We conducted a prospective evaluation (2024–2025) at a large academic medical center to evaluate the quality and instructional utility of AI-generated MCQs for clinical pathology. The evaluation compared performance across subspecialties using a uniform prompting protocol and a structured faculty review.

#### AI question generation

##### Model and configuration

All items were produced with OpenAI's GPT-4 (accessed via ChatGPT Pro). The model's knowledge was limited to content available up to June 2024. GPT-4 was accessed via the ChatGPT Pro web interface. When source documents were supplied (e.g. subspecialty references, College of American Pathologists (CAP)/Clinical Laboratory Improvement Amendments (CLIA) materials), prompts explicitly constrained generation to those sources to maintain accuracy and terminology consistency. Model specifications, control settings, and review governance are summarized in Table 1.

**Table 1:** A standardized prompt to generate multiple-choice questions (MCQs).

Element	Text used in this study
Standardized prompt	You are a board-style medical educator in clinical pathology. Using the provided resource(s), generate 10 multiple-choice questions (MCQs) with clinical vignettes. For each item: 1) Vignette near but not exceeding 257 characters 2) Provide 4 options (A–D) with one correct answer. 3) Keep answer choices similar in length; do not let the correct option be the longest. 4) Include a 1–3 sentence justification explaining why the correct option is correct and why the others are not. 5) Diversify item focus (diagnosis, methods, QC, informatics/regulation, interpretation); avoid repetitive “most likely diagnosis” stems. 6) If a source is supplied, base all content strictly on that resource; maintain terminology consistency and avoid PHI. 7) Output format: Question X: [vignette] A… B… C… D… Correct answer: [letter] Justification: [concise explanation].

The prompt was designed to produce items with consistent structure, length, and format.

### Prompting protocol

The standardized prompt (Table 1) was developed to align with the question format historically used in our weekly educational sessions. The prompt preserved the vignette-based MCQ structure familiar to faculty and residents, and incorporated character limits consistent with the online platform routinely used to distribute questions. Importantly, the vignette-based questions were initially designed to align with established best practices for one-best-answer item construction, drawing on the National Board of Medical Examiners (NBME) Item-Writing Guide, which our faculty previously used in local item-writing development efforts.^11^ This design emphasized pragmatic integration and conformed to contemporary standards for high-quality MCQs, allowing AI-generated items to fit seamlessly into established teaching workflows without disrupting session flow. The prompt was used across all sessions to promote consistency in item format and difficulty. The prompt instructed the model to “act as a board-style medical educator in clinical pathology,” required a clinical vignette, enforced strict length constraints (typically 250–257 characters), mandated four options (A–D) with a single best answer, and required a concise justification. The prompt further emphasized option-length parity to minimize cueing. Each session generated ten items.

### Source material integration

When applicable, reference materials were provided during prompt execution to constrain topic scope, terminology, and factual framing during question generation. These materials reflected the types of resources typically used by faculty when authoring assessment items, including subspecialty teaching resources, regulatory guidance documents, and institution-specific educational materials. Reference materials were used solely as contextual guidance for the model and were not reproduced verbatim in AI-generated outputs. All AI-generated questions underwent expert review and editing prior to inclusion in the analysis.

### Question review and expert panel

Items were evaluated by 19 pathology educators with relevant subspecialty expertise (board-certified attendings (13), fellows (3), and senior residents (3). Reviewer assignments were matched to subspecialty.

This study was intentionally designed as a pragmatic implementation within an existing weekly educational conference structure. Each session includes review of all clinical calls residents handled during the preceding week, a focused “chalk-talk”-style educational presentation, and real-time MCQ review. A single faculty member moderates the session and walks through each question with residents, providing answer justification and teaching points. AI-generated questions were incorporated into this established workflow without adding additional procedural steps or increasing faculty time burden.

In keeping with this design, most items were reviewed in advance by a single subspecialty-matched expert, typically the faculty member moderating that week's session, using a structured rubric. Across the study period, 19 raters participated using a unified rubric with shared operational definitions for question disposition (kept as is, kept with edits, discarded), clarity, and accuracy.

Differences in subspecialty counts reflect two primary factors: (1) targeted generation based on identified resident knowledge gaps, which varied by cohort and academic year, and (2) voluntary faculty participation in AI-assisted question drafting. Faculty were not required to use AI-generated items; adoption varied based on familiarity with LLM tools and existing reliance on established question banks.

### Rubric and disposition

Each item was scored using a standardized rubric with two domains—clarity and accuracy—each on a 1–5 scale. Reviewers then assigned a disposition: *kept as is*, *kept but edited*, or *discarded*. For items requiring revision or disposal, reviewers documented specific concerns (e.g. trivial content, inaccuracies, excessive complexity, insufficient alignment with learning objectives). See Supplemental Material 1.

### Data collection and quality assurance

Review data were captured in standardized spreadsheets that recorded subspecialty, domain scores, final disposition, and rationales for edits or discards. Before participation, reviewers received rating guidance to promote consistency.

### Statistical analysis

We summarized item outcomes (*kept as is, kept but edited, or discarded*) and domain scores using descriptive statistics overall and by subspecialty. Reviewer comments were analyzed qualitatively to identify recurrent themes in item design (e.g. clarity issues, content inaccuracies, and common cueing patterns). We calculated descriptive statistics (mean and standard deviation) for clarity and accuracy scores across all items. Independent samples t-tests were used to evaluate differences between items retained without revision versus those discarded, while one-way analysis of variance (ANOVA) was used to compare mean scores across subspecialties.

To evaluate how reviewer ratings influenced disposition decisions, we fit a proportional-odds (ordinal logistic) regression model with disposition as the ordered outcome (*discarded*, *kept but edited*, and *kept as is*). Clarity and accuracy ratings (both 1–5) were entered as predictors. Model estimation was performed using maximum likelihood with a logit link. Model adequacy was evaluated via log-likelihood, Akaike Information Criterion (AIC), and Bayesian Information Criterion (BIC). Predicted category probabilities were derived across the rating range and visualized as heatmaps and stacked probability plots to illustrate how incremental increases in rating scores shifted the likelihood of item retention.

Statistical analyses were performed in *Python* 3.9.12 using pandas and scipy, and statsmodels (OrderedModel).

### Qualitative analysis

We conducted a qualitative thematic analysis of reviewer feedback to capture broader insights into the strengths and limitations of AI-generated items. Comments were grouped post hoc into descriptive categories to summarize recurring exclusion and revision rationales, representing a pragmatic data-reduction exercise. This approach aligns with established qualitative content-analysis methods in applied health and educational research, which emphasize inductive categorization of textual data to identify common patterns.^12^ Similar qualitative content-analytic strategies have been used to categorize experiences and perceptions in diverse medical settings, such as patient and staff accounts of multidisciplinary interventions, organizational narratives about innovative care models, and patients’ descriptions of perioperative experiences.

### Hierarchical clustering of discarded questions

To systematically analyze the reasons for discarding questions, we conducted a qualitative content analysis of reviewer comments for all questions assigned a “*discarded*” disposition. Comments were coded and categorized into hierarchical clusters based on the nature of quality issues identified. Primary categories included educational appropriateness (encompassing difficulty calibration, board relevance, and educational focus), technical accuracy (including factual errors, answer choice problems, and disconnect from real-world practice), and strategic/curricular decisions (covering content gaps, redundancy, and scope limitations).

## Results

### Item generation and review outcomes

**Fig. 1:**
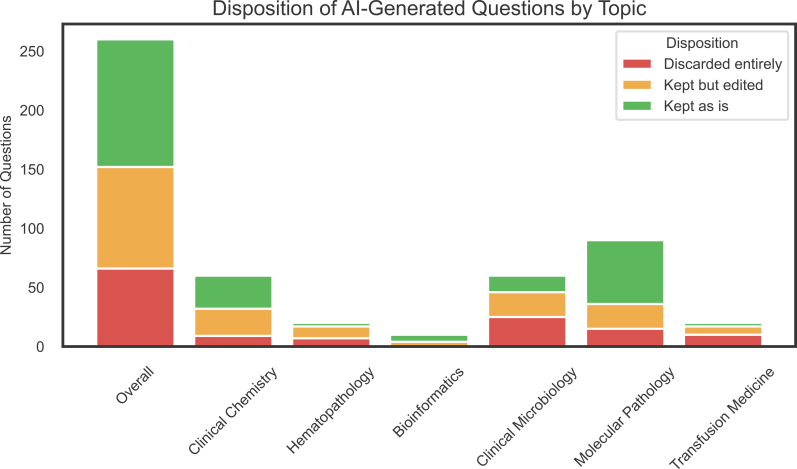
Disposition of AI-generated multiple-choice questions (MCQs) by subspecialty. Items were categorized as discarded entirely, retained without modification, or retained with edits following expert faculty review. Percentages are calculated within each subspecialty and overall. AI: artificial intelligence.

A total of 260 AI-generated MCQs were reviewed across six pathology subspecialties (Fig. 1). Reviewer dispositions were distributed as follows: *discarded entirely* (66/260, 25.4%), *kept but edited* (86/260, 33.1%), and *kept as is* (108/260, 41.5%). Subspecialty variation was evident, with molecular pathology producing the largest proportion of immediately usable items (kept as is: 54/90, 60%), while clinical microbiology yielded a relatively high discard rate (25/60, 41.7%).

### Reviewer ratings and disposition

Mean (SD) clarity and accuracy scores across all items were 4.5 (0.6) and 4.4 (0.9), respectively. Scores varied significantly by subspecialty for both clarity (*F* = 14.33, *P* < 0.001) and accuracy (*F* = 5.24, *P* < 0.001). For clarity, hematopathology items received the highest average ratings (5.0), while Informatics items received the lowest (3.6). For accuracy, molecular pathology items were rated highest (4.6), while informatics items were again the lowest (3.6). Disposition outcomes aligned strongly with these scores: items retained without revision (*kept as is*) had significantly higher mean clarity (4.8 vs 4.5, *P* < 0.001) and accuracy (4.6 vs 4.2, *P* < 0.05) scores compared with discarded items.

### Ordinal regression model

Proportional-odds modeling confirmed that clarity significantly predicted disposition. Each 1-point increase in clarity increased the odds of a more favorable disposition by 68% (*β* = 0.52, *SE* = 0.21, *P* = 0.013, odds ratio [*OR]* = 1.68, 95% confidence interval [*CI]:* 1.12–2.54). Accuracy showed a nonsignificant trend in the same direction, with each 1-point increase associated with 31% higher odds of a favorable disposition (*β* = 0.27, *SE* = 0.15, *P* = 0.066, *OR* = 1.31, 95% *CI:* 0.98–1.75). Threshold parameters were also statistically significant, including the cutpoint between *discarded entirely* vs *kept but edited* (*β* = 2.37, *SE* = 0.85, *P* = 0.005) and between *kept but edited* vs *kept as is* (*β* = 0.41, *SE* = 0.10, *p* < 0.001).

### Predicted probability profiles

**Fig. 2:**
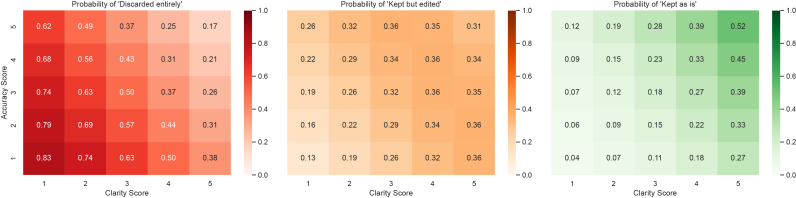
Predicted probabilities of question disposition by clarity and accuracy ratings. Heatmaps display model-based predicted probabilities of each disposition outcome (discarded entirely, kept but edited, kept as is) across the 1–5 range of Clarity (x-axis) and accuracy (y-axis). As both ratings increase, the likelihood of kept as is rises, while the likelihood of discarded entirely declines, consistent with monotonic behavior under the proportional-odds model.

**Fig. 3:**
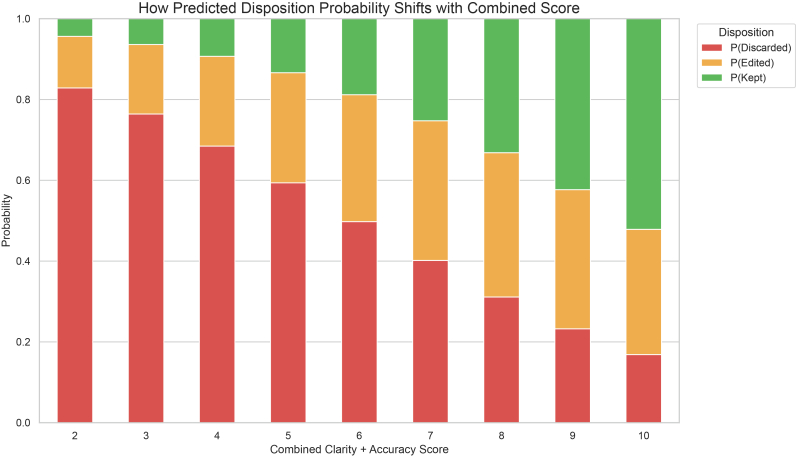
Shifts in predicted disposition probability by combined clarity + accuracy score. Stacked bar chart depicting the average predicted probability of each disposition outcome across the range of total scores (clarity + accuracy, 2–10). Higher combined scores are associated with progressively greater probability of kept as is and lower probability of discarded entirely, with kept but edited occupying an intermediate and narrowing range.

Model-derived predictions illustrated a clear gradient across the rating space. At the lowest ratings (clarity = 1, accuracy = 1), the probability of being *discarded entirely* was 85%, compared with only 3% for being *kept as is*. At the maximum ratings (clarity = 5, accuracy = 5), the probability of *kept as is* exceeded 50%, while discard probability fell below 20% (Fig. 2). When aggregated by combined clarity + accuracy score (range 2–10), results again showed monotonic trends (Fig. 3). Items with scores ≤4 were predominantly predicted to be discarded (>65%), while those scoring ≥9 had >75% predicted probability of being retained. The *kept but edited* category peaked in probability at total scores of 6–8, underscoring that mid to high-quality items were most often salvageable.

### Qualitative insights from reviewers

Reviewer feedback (see examples in Table 2) underscored a persistent tension between efficiency and rigor in AI-assisted item writing. On the one hand, many highlighted the streamlined production of well-formatted, exam-like questions: “*well written and ready to use without edits*,” one faculty member remarked, while another noted the benefit of having “*a template that feels like a board exam*.” Yet this ease sometimes came at the cost of distinctiveness or depth, with reviewers cautioning that items could feel “*too easy, also too similar to previous question*.”

**Table 2:** Key themes from qualitative feedback on AI-generated content.

Feedback Category	Description of issues identified	Selected reviewer comments (Quotes)
Clarity & question design	Feedback addressed ambiguous wording, poor-quality answer choices (distractors), and overall lack of clarity.	• “The question used abbreviations without defining them" • “Two answer choices were potentially correct" • “The incorrect choices were essentially the same thing"
Accuracy & clinical relevance	Comments pointed out factual errors, unrealistic scenarios, or content that did not align with real-world laboratory workflows.	• “Question answer totally incorrect as initially written" • “Newer consensus guidelines shifting … Discarded question because of difference in recommendations and real world application" • “Question did not make sense initially (did not represent usual workflow …)" • “Did not capture realistic workflow in lab" • “I disagreed with the correct answer. Edits … to make another choice the best option"
Scope & difficulty	Reviewers flagged items that were too easy, too difficult, or outside the intended educational scope for the target audience.	• “Question stem gives away the correct answer" • “Okay but too easy. Edits made to make it more challenging" • “Not helpful for residents … would rather have the question ask about appropriate testing" • “Topic slightly outside the scope of the quiz"

Reviewer comments were qualitatively analyzed and grouped into three distinct feedback categories. The table provides a description of the issues identified within each category, along with selected quotes that illustrate the primary concerns raised during the expert review process. AI: artificial intelligence.

Accuracy and logical coherence emerged as key stress points. Faculty frequently pointed to errors that, while subtle, undermined educational integrity. Comments such as “*correct answer not offered as a choice*” or “*choice C: erm41 is not a mutation*” exposed factual inaccuracies. Others identified reasoning flaws: “*the answer presumes a diagnosis that the stem never states*.”

Finally, reviewers raised broader considerations about fairness, pedagogy, and the evolving role of AI in assessment. Some flagged accessibility concerns—“*the question used abbreviations without definition*”—while others emphasized cognitive demand, warning that “*residents should be pushed to think more deeply, not just recall*.” Yet not all feedback was critical; efficiency was appreciated, with one expert writing, “*if I were giving the quiz again, I would make no edits*.”

### Hierarchical clustering of discarded questions

**Fig. 4:**
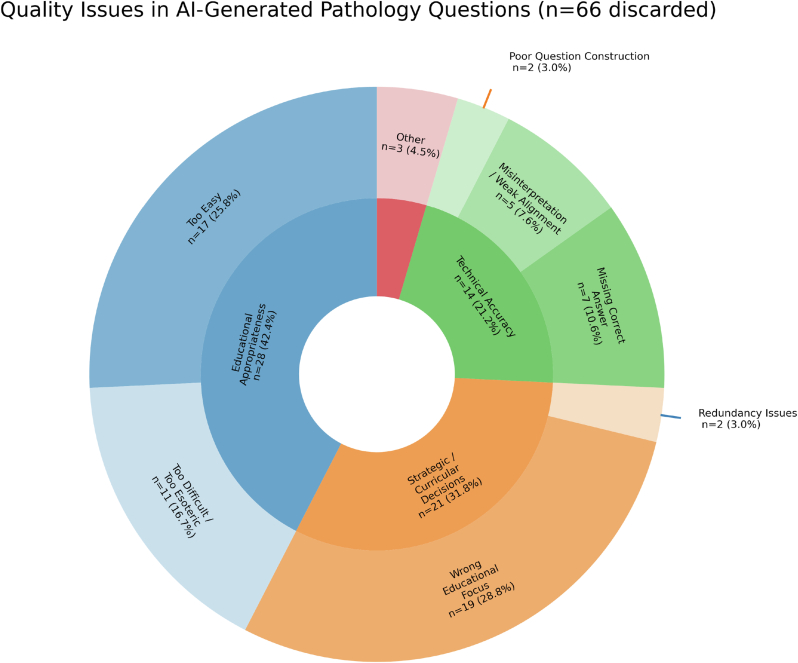
Reasons for discarding AI-generated pathology questions (n = 66). The inner ring shows the four main discard domains, whereas the outer ring shows subcategories. Segment area is proportional to count, and labels report n and % of all discarded questions. Colors denote domains, with lighter shades for subcategories. AI: artificial intelligence.

Analysis revealed (Fig. 4) that educational appropriateness issues were the most frequent concern, accounting for 42.4% of discards. Within this category, questions were most often judged too easy (25.8%), followed by too difficult or too esoteric (16.7%). Strategic and curricular decisions represented 31.8% of discarded items, driven largely by cases of incorrect educational focus (28.8%), with a smaller contribution from redundancy issues (3.0%). Technical accuracy problems comprised 21.2%, most commonly due to missing correct answers (10.6%), along with misinterpretation or weak alignment (7.6%) and poor question construction (3.0%). Finally, 4.5% of discards were classified as “Other”, reflecting instances where reviewers explicitly selected that option without additional explanation.

## Discussion

This study provides systematic evidence that LLMs can generate clinically relevant MCQs for clinical pathology, but with important caveats. Roughly four in ten items were immediately usable, and another third were salvageable with expert edits. Importantly, question clarity independently predicted question disposition, suggesting that measurable item qualities can guide triage of AI-generated outputs. Subspecialty-level variation also highlighted uneven performance, with molecular pathology showing relative strengths and clinical microbiology posing consistent challenges.

In addition to quantitative ratings, we used qualitative content analysis of reviewer comments to illuminate how subspecialty experts applied the evaluation rubric when deciding to discard AI-generated items. This pragmatic, category-based approach parallels qualitative content-analytic work in clinical and educational contexts, where prior investigators have used similar methods to structure patient and professional narratives about interventions, care models, and clinical experiences into recurrent domains for interpretation.^12^ As in prior studies that have categorized stakeholders’ experiences of multidisciplinary pain interventions, patient-centered organizational innovations, and perioperative care into recurring themes to inform practice development, our analysis summarizes how pathology educators weigh educational value, technical correctness, and curricular fit when integrating LLM-generated items into residency teaching. These qualitative categories complement the quantitative acceptance rates by clarifying the specific failure modes that most commonly lead to item exclusion, and they highlight which aspects of question quality may require the greatest faculty oversight as generative AI is incorporated into assessment workflows.

Our findings position generative AI not as a replacement for item writers but rather as an amplifier of expert capacity. The “*kept but edited*” category—where over one third of items landed—exemplifies this role. At the same time, AI functions as a stress test for assessment design. Subspecialty-specific weaknesses—such as lack of board relevance in clinical microbiology or inability to generate relevant images^13^ illuminate where model knowledge is shallow or diffuse. These gaps can inform targeted faculty oversight, directing attention toward domains where risks of inaccuracy or misrepresentation are greatest.^9^

Beyond evaluating AI-generated items, this study models a pragmatic framework for integrating AI into residency education. We introduce a structured paradigm of question disposition (kept as is, kept with edits, discarded) linked to standardized evaluative parameters such as clarity and accuracy. Importantly, AI-assisted drafting was embedded within an existing weekly educational workflow without increasing faculty burden or disrupting instructional structure. Residency programs adopting AI tools may benefit from designing implementation strategies that align with their own conference formats, faculty engagement patterns, and trainee knowledge gaps rather than pursuing uniform or externally imposed models.

Prior work in medical education has documented both enthusiasm and skepticism for LLM-assisted question generation.^2^^,^^14^ Studies in pharmacology,^15^ surgery,^7^ and general medical knowledge^16^ show moderate to high success rates, but emphasize that errors—particularly subtle inaccuracies—are common. Our discard rate of one in four is consistent with this literature, though the distribution across subspecialties adds a new dimension. The high acceptability of molecular pathology items may reflect that this field relies on codified variant-interpretation frameworks, such as the ACMG/AMP guidelines and subsequent disease-specific specifications, which translate heterogeneous evidence into rule-like criteria for classification and reporting^17^^,^^18^ that could be easier for LLMs to replicate. Conversely, clinical microbiology, with its reliance on complex workflows that integrate multiple diagnostic technologies, evolving resistance mechanisms, and region-specific epidemiology, challenges a model trained only on static text. Recent work evaluating LLMs for antibiotic prescribing and antimicrobial classification has shown that, although these systems can offer useful suggestions, they may overlook relevant agents, mishandle resistance patterns, and therefore require stringent human oversight to ensure clinically appropriate and safe recommendations.^19^^,^^20^

For pathology educators, the findings carry two major implications. First, AI may reduce the initial burden of item generation. Even when edits are needed, starting from a structured draft is plausibly more efficient than creating content *de novo*. Second, the high variability in quality means that institutions must design structured review pipelines and not simply rely on ad hoc oversight. In resource-constrained educational settings, this could expand assessment banks more rapidly^21^ and with less faculty fatigue.

Operationally, integrating AI-generated items may also alter faculty roles. Instead of acting primarily as authors, educators may increasingly function as curators and validators. This shift raises opportunities—freeing time for higher-order instructional design—and risks, such as erosion of faculty writing skills if over-reliance occurs. Maintaining a balance between AI-assisted and human-led content creation will therefore be essential.

This “human-in-the-loop” pattern mirrors recent work in health professions education, which argues that AI-assisted assessment is safest and most educationally valuable when faculty retain explicit responsibility for verifying content accuracy, guarding against bias, and aligning items with local curricula and assessment blueprints. Masters et al. highlight that LLMs should be embedded within transparent validation processes and governance structures, with faculty serving as accountable decision-makers rather than passive end-users.^1^ Blanco et al. similarly propose implementation roadmaps in which educators are trained to interpret and critically appraise AI outputs, and institutions clearly delineate who is responsible for monitoring errors and unintended consequences.^22^ Empirical comparisons of AI- versus human-generated MCQs further underscore that even when AI items approximate human questions in surface quality, expert review is needed to detect subtle inaccuracies, construct flaws, and misalignment with intended learning outcomes.^16^ Recent generative-AI frameworks for medical education extend these themes, recommending faculty development, explicit role definition, and continuous monitoring of AI tools so that efficiency gains do not come at the expense of pedagogical judgment or learner trust.^23^

A critical but often overlooked dimension of AI in medical education is alignment with regulatory and accreditation frameworks.^24^ CAP and CLIA set standards for laboratory quality and reporting that directly shape the content of pathology education. AI-generated items that fail to reflect current CAP/CLIA guidance risk not only inaccuracy but also misalignment with clinical practice standards. Similarly, the Accreditation Council for Graduate Medical Education (ACGME) emphasizes competency-based assessment in pathology residency. If AI-assisted questions are integrated into milestone evaluations, they must meet psychometric standards comparable to those traditional assessments to ensure fairness and reliability.^16^

Our findings suggest that while AI can accelerate item drafting, regulatory bodies will likely require robust evidence of validity before adoption in licensure or certification contexts.^3^^,^^25^ This creates an opportunity for collaboration: medical boards could leverage AI to expand item pools but only after multistage review that mirrors existing validation workflows. Importantly, transparency around AI use will be essential to maintain examinee trust. In this context, AI should be viewed as a supplementary tool within regulated ecosystems rather than an independent source of assessment content. Accrediting organizations may eventually issue guidance on how AI-generated items can be incorporated into formative versus summative settings, similar to how simulation-based assessments have been integrated over the past decade.^26^^,^^27^

Beyond technical accuracy, AI-generated items pose broader ethical challenges. Poorly designed questions can reinforce misconceptions, propagate bias, or create inequities in assessment if deployed without oversight.^14^ Additionally, the risk of overreliance on AI-generated content could inadvertently narrow curricular scope, if models preferentially generate items that align with well-documented topics at the expense of rare but critical content.^28^

At the policy level, integrating AI into medical education raises questions about intellectual ownership, accountability, and regulatory standards. Should accrediting bodies recognize the authorship of AI tools that drafted AI-assisted items? Should AI-generated content be subject to distinct psychometric validation before high-stakes use? As educational institutions adopt AI, these governance questions will become increasingly pressing.^29^

While our findings are informative, several limitations must be acknowledged. The evaluation was restricted to GPT-4 as accessed during the 2024–2025 academic year; performance may differ with newer LLM versions or domain-specific fine-tuning. Additionally, although a standardized prompt was used to standardize input structure (vignette format, length, and pedagogical cues), outputs remain probabilistic and can vary across runs. Reviewer subjectivity, despite rubric training, may have influenced ratings, particularly for borderline items. There is also considerable variation in reviewer experience, ranging from faculty members with nearly 30 years of expertise to 4th year residents and fellows still in training. We also note that some subspecialties, such as hematopathology, transfusion medicine, and informatics, were represented by relatively few questions in this study. Many of these items were generated intentionally to target specific knowledge gaps identified in the current resident cohort. This design choice likely influenced the distribution of questions across subspecialties and may limit generalizability of subspecialty-level performance comparisons.

Although a unified rubric with shared operational definitions was used across all 19 raters, individual reviewers may apply different thresholds for acceptance even when using shared criteria, a recognized challenge in expert panel–based evaluation.^30^^,^^31^ This effect may be amplified in subspecialties with limited domain expertise, where the perspectives of a small number of reviewers disproportionately influence aggregate outcomes.^32^ Accordingly, subspecialty differences in disposition rates should be interpreted cautiously, as they may reflect both true variation in AI-generated item quality and reviewer-specific standards for acceptance. Additionally, we did not evaluate learner-facing outcomes such as test performance, item discrimination indices, or long-term knowledge retention. Such analyses will be critical to determining whether AI-generated items are not only usable but pedagogically effective.

Future research should extend to longitudinal trials comparing learner performance with AI-assisted versus human-authored assessments. Cross-model comparisons—including open-source and fine-tuned domain-specific LLMs—may identify configurations with greater reliability. Finally, integrating real-time reference updates (e.g. continuous ingestion of CAP/CLIA standards) may help mitigate some of the technical accuracy issues underlying a subset of the discarded items.

## Conclusions

Generative AI is capable of producing clinically relevant questions in clinical pathology, but its outputs remain uneven. Approximately three-quarters of items were ultimately ueable, either directly or with edits, supporting AI's role as a powerful accelerator in assessment development. However, consistent expert oversight is indispensable, particularly in knowledge domains where precision and recency are paramount. The path forward lies in building hybrid workflows where AI provides structured drafts and faculty provide the rigor, nuance, and accountability. Done responsibly, this approach may not only relieve item-writing bottlenecks but also expand the adaptability, relevance, and fairness of pathology education—while ensuring alignment with CAP, CLIA, and ACGME competency requirements.

## Declaration of generative AI in scientific writing

The authors utilized ChatGPT (OpenAI) to assist with language editing/polishing during manuscript preparation. The authors reviewed and edited the content as needed and take full responsibility for the content of the publication.

## Funding

The article processing fee for this article was funded by an Open Access Award given by the Society of ‘67, which supports the mission of the Association for Academic Pathology to produce the next generation of outstanding investigators and educational scholars in the field of pathology. This award helps to promote the publication of high-quality original scholarship in *Academic Pathology* by authors at an early stage of academic development.

## Declaration of competing interest

The authors declare that they have no conflicts of interest to disclose.
