# Supplementary material for: Evaluating artificial intelligence-generated multiple-choice questions in clinical pathology
Source: Acad Pathol. 2026 Jun 17;13(3):100275. doi: 10.1016/j.acpath.2026.100275 (PMC13311378; doi:10.1016/j.acpath.2026.100275)
Supplement: Multimedia component 1 [file mmc1.pdf]

Supplemental Material 1

Faculty Evaluation of AI Questions

	Question Disposition (Discarded entirely, kept as is, kept but edited)	Clarity	Accuracy	If Discarded, Reason for Discard	Additional Comments (optional)
**Question 1**					
**Question 2**					
**Question 3**					
**Question 4**					
**Question 5**					
**Question 6**					
**Question 7**					
**Question 8**					
**Question 9**					
**Question 10**					

Clarity

1: Very Confusing - The quiz question is extremely unclear, difficult to understand, and contains significant ambiguity.

2: Somewhat Confusing - The quiz question has several unclear elements and may cause confusion, though parts of it might be understandable with effort.

3: Moderately Clear - The quiz question is somewhat clear but has some ambiguous parts that could be misinterpreted.

4: Mostly Clear - The quiz question is mostly clear and easy to understand, with only minor ambiguities.

5: Very Clear - The quiz question is perfectly clear, easy to understand, and free of any ambiguity.

Accuracy

1: Completely Incorrect - The quiz answer is entirely incorrect and not confirmed by a pathologist, containing major factual errors.

2: Mostly Incorrect - The quiz answer has significant errors and is largely not confirmed by a pathologist, with only a few correct elements.

3: Partially Correct - The quiz answer is partially correct but contains notable inaccuracies. A pathologist might recognize some correct elements but would still need to correct several points.

4: Mostly Correct - The quiz answer is mostly correct with minor errors. A pathologist would generally agree with the answer but might suggest slight modifications.

5: Completely Correct - The quiz answer is entirely correct and confirmed by a pathologist, with no errors or inaccuracies.
